# Personalized Nutrition in the Pediatric ICU: Steering the Shift from Acute Stress to Metabolic Recovery and Rehabilitation

**DOI:** 10.3390/nu16203523

**Published:** 2024-10-17

**Authors:** George Briassoulis, Stavroula Ilia, Efrossini Briassouli

**Affiliations:** 1 Postgraduate Program “Emergency and Intensive Care in Children Adolescents and Young Adults”, School of Medicine, University of Crete, Section 6D (Delta), Office 03, Voutes, 71003 Heraklion, Greece; stavroula.ilia@uoc.gr; 2 Paediatric Intensive Care Unit, University Hospital, School of Medicine, University of Crete, 71110 Heraklion, Greece; 3 Infectious Diseases Department “MAKKA”, “Aghia Sophia” Children’s Hospital, First Department of Pediatrics, School of Medicine, National and Kapodistrian University of Athens, 11527 Athens, Greece; efroelesar@hotmail.com

**Keywords:** nutrition, critical illness, intensive care, children, indirect calorimetry, macronutrients, energy, protein, micronutrients

## Abstract

Background: Nutrition significantly impacts the outcomes of critically ill children in intensive care units (ICUs). Due to the evolving metabolic, neuroendocrine, and immunological disorders associated with severe illness or trauma, there are dynamically changing phases of energy needs requiring tailored macronutrient intake. Objectives: This study aims to assess the changing dietary needs from the acute phase through recovery, provide recommendations for implementing evidence-based strategies to ensure adequate energy and nutrient provision in pediatric ICUs, and optimize patient outcomes. Methods: A comprehensive search of the MEDLINE-PubMed database was conducted, focusing on randomized controlled trials, meta-analyses, and systematic reviews related to the nutrition of critically ill children. The study highlights recent guidelines using the GRADE approach, supplemented by relevant adult studies, current clinical practices, challenges, gaps in knowledge, and future directions for research aimed at improving nutritional interventions. Results: Early personalized, incremental enteral feeding helps mitigate the negative energy balance during the acute phase, aids organ function restoration in the stabilization phase, and supports growth during the recovery phase and beyond. Conversely, early full nutritional support, high protein doses, or isolated micronutrient administration have not demonstrated benefits due to anabolic resistance in these patients. Moreover, early parenteral nutrition during the acute phase may suppress autophagy and lead to worse outcomes. Accurate assessment of nutritional status and monitoring of daily energy and protein needs are crucial. Conclusions: Strong evidence supports the establishment of a dedicated nutritional team and the implementation of individualized nutritional protocols in the ICU to reduce morbidity and mortality in critically ill children.

## 1. Introduction

Although optimal nutrition is considered vital for improving outcomes in critically ill children, there is a notable lack of high-quality evidence to guide nutritional practices in pediatric intensive care units (PICUs). The shortage of robust scientific data on nutrition in PICUs from well-designed randomized controlled trials (RCTs) has resulted in diverse nutritional approaches globally [1]. In 2017, the American Society of Parenteral and Enteral Nutrition (ASPEN) and the Society of Critical Care Medicine (SCCM) released updated guidelines [2]. In 2020, the European Society of Pediatric and Neonatal Intensive Care (ESPNIC) revised these guidelines for providing nutritional support to critically ill pediatric patients [3].

The metabolic response to critical illness progresses through distinct sequential phases, each with different energy requirements, necessitating personalized metabolic and nutritional support through medical nutrition [4]. The acute phase, characterized by vital organ failure and the need for organ support, usually lasts three or more days and is associated with decreased energy expenditure. As patients transition to the stable phase, marked by the stabilization of organ function and the gradual reduction in support, their resting energy expenditure normalizes. The recovery phase follows, featuring clinical improvement and increased patient activity, which demands higher energy and protein intake to meet the increased needs of recovery and mobilization.

## 2. Methods

The MEDLINE-PubMed database was chosen to search for full-text publications and English-language journals from 2004 to 2024, with an emphasis on studies published within the last three years. The search included guidelines, randomized placebo-controlled trials (RCTs), controlled clinical trials, double-blind, randomized controlled studies, meta-analyses, and systematic reviews. The following combinations of keywords were used: nutrition OR nutrition guidelines AND critical illness OR intensive care OR pediatric OR indirect calorimetry OR macronutrients OR micronutrients OR energy OR protein. Additionally, we conducted a manual search of the reference lists of the selected studies. When pediatric studies were lacking, limited, or of poor quality on a particular topic, evidence from adult randomized controlled trials was used to fill the knowledge gap.

Population, intervention, comparison, results, and study design (PICOS) are shown in the Supplementary Material (Table S1). By including a variety of studies, authors’ judgments about each risk of bias item for each included study were impossible. Although selection biases cannot be excluded, this review was built on the foundation of all recent guidelines of nutrition for critically ill patients, which were supported exclusively by selected RCTs (Table S2). Guidelines avoided biases using a Grading of Recommendations, Assessment, Development, and Evaluations (GRADE) approach to assess the quality of the study design and execution (Table S3).

## 3. Metabolism in Critical Illness

**Figure 1:**
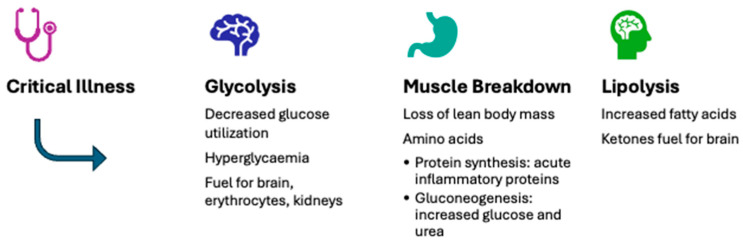
Typical metabolic adaptations during the acute phase of severe illness.

Severe illness triggers major metabolic and endocrine disturbances that closely interact with changes in the autonomic and immune systems. During the acute phase, there is increased catabolism, insulin resistance, and changes in nutrient substrate utilization [4]. These adaptations help ensure cellular energy sufficiency, support the body’s defense mechanisms, and promote survival (Figure 1). However, these disturbances can become harmful after the acute phase [5]. Critically ill children often experience feeding difficulties due to perceived feed intolerance and interruptions [6], resulting in undernourishment and a cumulative macronutrient deficit during their PICU stay [7].

### 3.1. Catabolism

Severe mitochondrial dysfunction related to critical illness leads to a depletion of adenosine triphosphate (ATP), which is believed to contribute to organ failure [8,9]. To restore energy, the body enters an acute phase, where catabolic reactions breakdown nutrient substrates to generate energy. These reactions breakdown large organic molecules into smaller ones, releasing the energy stored in their chemical bonds. Approximately 40% of this energy is converted into ATP, the primary energy currency of cells, while the remaining 60% is dissipated as heat, which is absorbed by tissues and body fluids. Because this vital endogenous energy production cannot be reversed, accurately determining macro- and micro-nutrient requirements during the catabolic phase of critical illness is difficult. Studies suggest that providing full nutrition during this phase does not counteract catabolism and may even increase the metabolic burden, potentially causing harm [10,11]. On the other hand, insufficient energy and protein intake can exacerbate catabolism, leading to muscle loss, weakness [12], infections, slower recovery, and higher mortality [13].

### 3.2. Anabolism

During the anabolic phase, when nutritional requirements significantly increase to facilitate recovery and catch-up growth, energy-consuming chemical reactions (ATP breakdown) build larger molecules from smaller ones. This process powers the molecular machinery responsible for constructing new cells and tissues, repairing damaged tissues, absorbing and storing nutrients, contracting muscles, and generating electrical potential in nerve cells. During the recovery phase of pediatric critical illness, also known as catch-up growth, muscle recovery and growth necessitate nutritional intake of at least twice the resting energy expenditure [5]. Considering these large intake demands, an alternative approach is needed, particularly when intestinal issues hinder full enteral feeding. In addition to nutritional recovery, mobilization and exercise are essential for promoting catch-up growth and attaining optimal body composition [5].

## 4. Assessment of Nutritional Status

Due to the dynamic metabolic and endocrine responses during critical illness, nutritional support should be tailored to these changes and adjusted across different phases of pediatric critical illness. Both ASPEN [2] and ESPNIC [3] recommend evaluating the nutritional status of all critically ill children upon admission and weekly during their stay in the PICU. Specifically, it is advised to:

Conduct anthropometric measurements at admission and at least weekly throughout hospitalization, as patients are at risk of nutritional decline, which can negatively impact outcomes.

Express these measurements in z-scores, including weight, height/length, body mass index for age (weight for length in children under two years), mid-upper-arm circumference, and head circumference in neonates and infants. This helps identify patients at the extremes who may benefit most from early nutritional assessment and interventions.

### 4.1. Malnutrition

Malnutrition is common among mechanically ventilated children at the time of PICU admission. Upon admission, over one-third of critically ill children are malnourished [14]. Malnutrition is linked to an increased risk of mortality, a higher incidence of hospital-acquired infections, fewer ventilator-free days, and a reduced likelihood of discharge from the hospital [15]. In Europe, malnutrition at PICU admission ranges from 15% to 25% [16,17,18]. Malnourished hospitalized patients experience higher complication rates, increased mortality, longer hospital stays, and higher hospital costs. In the United States, a retrospective analysis of 4106 children admitted to PICUs found that 1 in 5 had a diagnosis of malnutrition. These patients were younger, often had comorbidities, experienced longer hospital stays, had higher 30-day readmission rates, and utilized more healthcare resources [19]. In India, 51.2% of critically ill children were malnourished, with an overall mortality rate of 38.8% [20]. In Ethiopia, the prevalence of wasting in a PICU was 37.8%, and stunting was 45.7% [21]. Malnourished children were more likely than their well-nourished peers to require mechanical ventilation, spend more time on it, develop hospital-acquired infections more frequently, and have prolonged hospital stays [21]. Therefore, targeted strategies to prevent malnutrition in this overlooked group should be incorporated into current healthcare systems and nutrition programs.

### 4.2. Nutritional Deterioration

Critical illness can also exacerbate nutritional deterioration, leading to poor outcomes [22]. Thus, a significant number of critically ill children admitted to PICUs globally experience nutritional deficiencies [17,20,23,24,25]. Early deterioration of nutritional status is common, with nearly one-third of critically ill children experiencing a decline in their nutritional indices [26]. This occurs regardless of pre-existing malnutrition, as the combination of metabolic disturbances and the inability to meet basic nutritional needs often leads to undernutrition, resulting in a cumulative macronutrient deficit during the PICU stay [3]. In Brazilian PICUs, 23% of children showed nutritional status deterioration, often associated with complex chronic conditions (CCCs) [25]. After discharge from five tertiary Canadian PICUs, 24.1% of patients experienced complications, and 19.5% were readmitted to the hospital. The likelihood of complications and readmission was nearly three times higher for those at high nutritional risk or identified as malnourished [27].

### 4.3. Muscle Mass Loss

During the critical stages of illness, muscle mass loss is rapid, severe, and ongoing. Prolonged stays in the intensive care unit increase the risk of muscle weakness, which can lead to life-threatening complications, such as difficulties in weaning from the ventilator and swallowing problems [12]. For those who survive critical illness, this persistent weakness is now recognized as part of post-intensive care syndrome [28].

The updated Global Leadership Initiative on Malnutrition (GLIM) criteria for malnutrition emphasize the importance of reduced muscle mass as a key factor in diagnosing malnutrition across all patients [29]. This muscle mass loss can also be identified through an elevated urea-to-creatinine ratio, an early marker of the catabolic state often seen in extended critical illness [30]. Studies using computed tomography (CT) scans have shown a significant correlation between the reduction in iliopsoas muscle size at the L3–L4 vertebral level and the duration of ICU stays [30]. Although CT scans provide standardized muscle mass measurements with low variability, repeated CT scans are impractical due to high costs, radiation exposure, and logistical challenges.

Muscle ultrasound has recently gained attention as a method to measure catabolism and is being used as an outcome measure in interventional studies [31]. In a prospective validation study conducted in a trauma-focused ICU, ultrasound-based measurements of femoral quadriceps muscle thickness showed performance comparable to that of thigh CT [32]. Its benefits include easy access, minimal risk and cost, and the ability to detect conditions such as necrosis and fasciitis, along with its established link to physical function both inside and outside of the ICU. In critically ill COVID-19 patients, combined ultrasound revealed distinct changes in muscle thickness for both the diaphragm and the rectus femoris, with the most significant muscle loss occurring between days 5 and 10. This reduction in muscle thickness was associated with functional outcomes, as only 31% of patients were able to return to their pre-admission residence without requiring additional rehabilitation [33]. However, the technique lacks standardization, and image acquisition and analysis can vary, which may result in small changes in muscle mass going undetected [34].

## 5. Energy Requirements

Accumulated energy deficits during the acute phase of critical illness can lead to adverse clinical and nutritional outcomes. Multiple observational studies have linked increased nutritional intake with improved outcomes in critically ill patients [35,36]. According to the updated ASPEN guidelines, it is essential to carefully evaluate individual energy needs and timely initiate appropriate energy targets to avoid unintentional caloric deficits or excesses [2].

### 5.1. The Acute Phase

In the acute phase of severe illness, the body’s endogenous energy production can supply about 50% to 67% of the total energy requirements, regardless of the external energy intake [37]. Consequently, in critically ill children, energy intake should not exceed their basal metabolic rate and should remain below their resting energy expenditure during this period. It is recommended to limit energy intake to a maximum of 70% of the patient’s energy expenditure, as internal energy production accounts for at least 30% of their basal metabolic rate [2]. Meeting approximately 70% of energy requirements during the first week of the ICU stay was linked to lower 28-day mortality in mechanically ventilated, critically ill patients, particularly those who were at high nutritional risk upon admission [38]. In adults, providing 100% of the recommended calorie intake during critical illness, compared to 70%, did not significantly improve quality of life, functional outcomes, or survival rates after six months [39]. Observational studies suggest that ICU patients should receive at least two-thirds of their estimated daily energy needs by the end of the first week [2]. Additionally, some evidence suggests that providing more than 80% of the energy expenditure may be associated with higher mortality, although recent large trials found no significant difference in mortality between those receiving 22 and 30 kcal/kg/day [40]. A recent meta-analysis confirmed that early full feeding in critically ill patients is more harmful compared to permissive underfeeding [41]. The 2022 ASPEN guidelines for adults recommend meeting basal metabolic needs by providing 12–25 kcal/kg/day during the first seven days in the ICU [42]. A moderate daily intake of 10–20 kcal/kg and 0.8–1.2 g/kg of protein, as opposed to a lower intake (energy < 10 kcal/kg, protein < 0.8 g/kg), has been associated with a higher likelihood of successful weaning from ventilation and a lower risk of death in the ICU [43].

### 5.2. The Recovery Phase

In the recovery phase, after the hypometabolic state, energy intake should be increased to offset energy deficits, support physical activity, aid in recovery, and promote growth, leading to a hypermetabolic state [44]. During the recovery phase following severe trauma or critical illness, achieving catch-up growth and muscle recovery requires an energy intake that is at least twice the resting energy expenditure [5]. While the acute phase predominantly involves carbohydrate metabolism, the recovery phase sees a more balanced utilization of various nutrient substrates [5]. Persistent hypometabolism in critically ill ICU patients often signals an irreversible energy deficiency and is a poor prognostic indicator [45]. ICU survivors often experience prolonged physical disabilities, particularly if they were on a ventilator for more than 48 h or had significant multiple organ failure (MOF). Therefore, personalized nutrition and exercise plans are critical throughout the patient’s ICU journey [46].

### 5.3. Post-PICU Discharge

**Figure 2:**
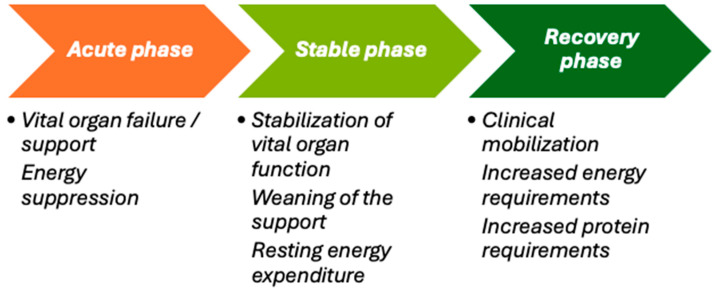
The evolving energy demands during different metabolic phases of critical illness.

Nutritional support provided during the ICU stay should continue after PICU discharge [47] to prevent ongoing or worsening nutritional deficits during recovery [48]. Unfortunately, nutritional care often deteriorates after ICU discharge. Implementing a structured nutrition delivery strategy can improve outcomes, as highlighted in a recent review on ICU and post-ICU nutrition [49]. A well-designed algorithm for hospitalized patients at high nutritional risk has shown significant reductions in mortality and complications within 30 days, along with improved recovery and functional independence [50]. Personalized exercise programs are becoming increasingly important in ICU recovery research (Figure 2). Considering the unsatisfactory results of existing adult ICU rehabilitation trials that used a one-size-fits-all approach, future ICU rehabilitation may benefit from personalized exercise programs guided by cardiopulmonary exercise testing (CPET) [51].

## 6. Energy Expenditure Measurement

During acute critical illness, patients often undergo feeding-resistant catabolism and significant insulin resistance, particularly in the liver. This condition inhibits the suppression of endogenous glucose production and energy generation, even with the provision of nutrients and insulin [52]. Therefore, administering extra calories based on resting energy expenditure (REE) during the early acute phase, without inhibiting gluconeogenesis, may exacerbate hyperglycemia and hypertriglyceridemia, further burdening the liver [53]. Observational studies suggest that current guidelines recommend including a nutrition support team, with a specialized dietitian, in the PICU team to ensure timely nutritional assessments, accurate energy expenditure calculations, and optimal nutrient delivery and adjustments for the patients [2].

### 6.1. Indirect Calorimetry

Indirect calorimetry (IC) is the only tool currently available for accurately measuring a patient’s energy expenditure in routine clinical settings, allowing for personalized nutrition therapy [54]. In cases of acute illness, IC is considered the gold standard for measuring resting energy expenditure (REE) in children in the PICU [2,3]. Modern metabolic monitors used for IC measure airflow via pressure or heat differences just after the endotracheal tube, using a pneumotachograph. These monitors synchronize airflow and air sample measurements with oxygen consumption (Vo_2_) and carbon dioxide production (Vco_2_) on a breath-by-breath basis, averaging values per minute. The patient’s daily energy expenditure is then calculated using the modified Weir equation: REE = (Vo_2_ (3.94) + Vco_2_ (1.11)) × 1440 [55]. Additionally, the respiratory quotient (RQ) helps identify underfeeding (RQ < 0.7), technical issues (like leaks), or overfeeding (RQ > 1.0). It has also been demonstrated that only Vo_2_ and Vco_2_, not IL-6 or IL-10, were linked to a hypometabolic pattern that was prevalent during the acute phase of stress and associated with higher mortality [56].

In children, energy needs should ideally be measured using IC [45]. Although randomized controlled trials have not consistently shown a clear advantage of IC [57,58], a recent meta-analysis suggested a potential reduction in mortality when IC-guided feeding was initiated within the first week of critical illness in adults, compared to feeding based on calculated energy targets [59,60]. Similarly, a systematic review and meta-analysis of four prospective randomized trials found a decrease in 28-day mortality when IC-guided isocaloric nutrition was compared to hypocaloric nutrition in critically ill adults [61]. In a study of 139 critically ill children, those who were overfed had significantly longer stays in the PICU and hospital compared to those who were fed according to measured energy requirements [45].

A key retrospective study involving about 1200 ICU patients found that delivering around 70% of REE was associated with improved survival in the ICU [62]. Both underfeeding and overfeeding beyond 80% of IC targets were linked to higher mortality during the acute phase of ICU care [62]. Other studies using IC have suggested that individualized nutrition can reduce infectious complications and lower costs, although this approach has not yet been tested in sufficiently large randomized controlled trials [63]. Unfortunately, no bedside monitor currently exists to measure the amount of endogenous substrate mobilized, making it difficult to assess the duration and extent of un-suppressible endogenous substrate production in individual patients [64].

Therefore, while IC-guided nutritional targets and REE measurements may be critical for future personalized ICU nutrition strategies, IC data should be used carefully. As a result, current ESPEN guidelines do not always recommend matching energy expenditure measured by IC with feeding targets in critically ill adult patients [65]. Most recent ICU nutrition guidelines suggest starting energy delivery at about 10–15 kcal/kg or less than 70% of the measured IC REE, whether enteral or parenteral nutrition is used, and gradually increasing as the patient stabilizes [2,42,66]. In centers with trained staff, including dietitians, using IC-measured REE in appropriate patients can help prevent the common issues of over- or under-feeding in the ICU [62]. This can provide a more focused and objective approach to nutrition therapy, which plays a crucial role in recovery during and after ICU stays [49]. In the post-ICU setting, it is important to consider ongoing use of IC and other muscle assessments to guide nutritional support.

### 6.2. Predictive Equations

In most clinical settings, the lack of indirect calorimetry devices leads to using prediction equations, such as Schofield’s (which considers age, gender, and weight), to estimate resting energy expenditure (REE) in PICUs [67] without incorporating stress factors [68]. However, the Harris–Benedict equations and the Recommended Dietary Allowances (RDAs) from the Dietary Reference Intakes are unsuitable for determining energy needs in critically ill children [2]. Recent cohort studies in critically ill children have demonstrated that 16 published predictive equations for energy consumption [18], or those relying solely on Vco_2_ measurements with a fixed RQ value [69], are inaccurate and may result in unintentional overfeeding or underfeeding [70]. Similarly, predictive equations have proven unreliable for estimating REE in adult ICUs across 12 equations [71,72]. Additionally, recent data indicate that the metabolic rate measured by indirect calorimetry in COVID-19 patients significantly deviates from values predicted by commonly used equations [73,74].

## 7. Macronutrient Requirements

Monitoring tools that accurately quantify the actual macronutrient need for the individual patient are currently not available [13]. Recent research has shown that during the acute phase of critical illness, high doses of all macronutrients should be avoided due to anabolic resistance and the potential risks of inhibiting cellular repair processes, as well as increasing hyperglycemia and the need for insulin. Without access to a metabolic monitor and considering the insights mentioned, it may be wise to temporarily reduce macronutrient intake in patients facing a new severe event in the ICU, such as the onset of septic shock during their stay [75].

### 7.1. Protein

In the acute phase of critical illness, children experience increased protein turnover, characterized by heightened protein synthesis and breakdown throughout the body. This leads to a net-negative protein balance and muscle wasting, which can negatively impact outcomes [76]. Muscle wasting often persists during the recovery phase due to immobilization and undernutrition [37]. Although, increasing protein intake through enteral or parenteral nutrition does not prevent or reverse muscle protein breakdown, and it may even harm muscle structure [77] and function [78]. Moreover, supplementing insufficient enteral nutrition with parenteral nutrition early on can raise the muscular fat content, exacerbate muscle weakness, and impede recovery [78]. Even low doses of parenteral amino acids in the first week of the PICU stay have been associated with longer hospital stays, prolonged mechanical ventilation, and a rise in new infections [79]. Secondary analyses of the EPaNIC and PEPaNIC RCTs suggest that the harm linked to early parenteral nutrition is due to amino acid doses rather than glucose or lipid doses [79,80].

Guidelines from SCCM and ASPEN recommend providing critically ill children with 1.5 g/kg of protein daily through enteral feeding to avoid a negative nitrogen balance [2]. Similarly, ESPNIC advises a minimum enteral protein intake of 1.5 g/kg/day to prevent negative protein balance [3]. The European ESPEN guidelines for critically ill adults recommend gradually administering 1.3 g/kg of protein [81]. Despite guidelines advocating for higher amino acid intake during parenteral nutrition [82], no scientific evidence supports additional protein or amino acid intake during the acute illness phase [83]. Low-dose protein delivery should be used early (days 1–2: <0.8 g/kg/day) and progress to ≥1.2 g/kg/day as patients stabilize, with consideration of avoiding higher protein intake in unstable patients and acute kidney injury [75]. Providing more than 1.2 g/kg of protein to critically ill patients appeared to enhance the nitrogen balance, positively impact short-term muscle mass changes, and potentially reduce 60-day mortality [84]. Administering higher protein doses (≥2.2 g/kg/day) compared to standard doses (≤1.2 g/kg/day) in critically ill patients on mechanical ventilation did not improve time-to-discharge-alive and resulted in worse outcomes for those with acute kidney injury and high organ failure scores [85]. A high protein intake (≥2.2 g/kg/day) was linked to potentially harmful effects across all stages of acute kidney injury, particularly in patients not receiving kidney replacement therapy [86]. Similarly, a nephroprotective RCT involving 474 critically ill adults found no benefit from early amino acid supplementation at approximately 1.75 g/kg/day during the ICU stay and observed a significant increase in ureagenesis [87]. In an international, multicenter, pragmatic, registry-based randomized trial in critically ill patients with obesity, increasing the protein intake did not lead to better clinical outcomes, even in those at higher risk due to nutritional deficiencies or frailty [88]. Other RCTs revealed that early full feeding significantly increased ureagenesis, even with relatively low amino acid doses (around 0.8 g/kg/day) [79,89,90]. Furthermore, higher protein intake was linked to increased muscle wasting, inhibition of autophagy, enhanced ureagenesis, prolonged organ failure, and longer hospital stays [89]. Importantly, even when protein preserves muscle mass, this does not always correlate with improved muscle function or strength [91].

#### 7.1.1. Muscle Protein Synthesis

It is crucial to recognize that increased amino acid provision may not enhance muscle protein synthesis during the acute phase. A recent study found that ICU patients exhibited 60% less muscle protein synthesis compared to healthy individuals, despite normal gut protein absorption [92]. The blunted anabolic response in ICU patients may result from factors such as anabolic resistance (reduced effects of protein and exercise on muscle protein synthesis), immobilization, insulin resistance, inflammation, decreased satellite cell numbers, and low muscle ATP levels [93]. Notably, a recent meta-analysis showed that combining early mobilization with early nutrition significantly impacted the length of stay in intensive care and the hospital and positively affected functionality and quality of life [94].

#### 7.1.2. Protein Calculation

Total body weight (TBW) is generally used to calculate the protein dosage, though calculations based on lean mass (LM) might be preferable [75]. Bedside tools, such as bioimpedance assessment (BIA), can estimate LM, with alternatives including muscle ultrasound and CT scans [95]. Currently, no specific, validated biomarkers for muscle breakdown, autophagy, inflammation, or insulin resistance exist to identify patients who might benefit from a higher protein intake. While some studies suggest that achieving a positive nitrogen balance through a higher protein intake is associated with better outcomes [45], other research shows that an increased protein intake may lead to enhanced ureagenesis and urinary nitrogen loss, raising questions about whether higher intake directly promotes muscle protein synthesis [96]. The elevated risk of death observed in patients assigned to a higher protein dose in the EFFORT Protein trial was thought to be mediated by increased urea cycle activity, with serum urea serving as a biological marker. Consequently, it was recommended that serum urea levels be considered when starting and adjusting protein delivery in critically ill patients [97].

### 7.2. Glucose

Intravenous glucose solutions should be administered in adequate amounts to prevent hypoglycemia (below 45 mg/dL) [98]. However, it is equally important to avoid hyperglycemia (above 145 mg/dL), which is associated with increased mortality rates, enhanced fat production and storage, and the potential development of liver steatosis [99]. If blood glucose levels consistently exceed 180 mg/dL, continuous insulin infusion should be initiated to manage the levels. When medical nutrition results in a respiratory quotient (RQ) greater than 1.0, coupled with issues in glycemic control or respiratory acidosis, the feedings should be reduced [42].

### 7.3. Lipids

During illness and physiological stress, lipid metabolism generally increases, making lipids a crucial energy source. Providing parenteral lipids after the first week of hospitalization can offer a significant energy supply without requiring large amounts of carbohydrates. To prevent essential fatty acid deficiencies, a minimum intake of 0.1 g/kg/day of linoleic acid is recommended [100]. Current guidelines suggest that the dosage of parenteral lipids should not exceed the body’s capacity to clear lipids, with a maximum of 4 g/kg/day for adults and 3 g/kg/day for infants and children [3,101]. For parenteral nutrition, synthetic lipid emulsions, with or without fish oil, are recommended as the preferred option. If medical nutrition leads to elevated serum triglyceride levels, feedings should be reduced. Additionally, lipid-based sedation contributes to the total energy intake and should be factored into the overall daily energy calculations [42].

## 8. Micronutrients

The initiation of medical nutrition can alter cellular or tissue levels of micronutrients, leading to shifts between compartments during critical illness [102]. Recommendations for maintaining baseline needs in adults are currently based on ESPEN micronutrient guidelines [103]. Recent ESPEN guidelines highlight the essential roles of micronutrients, especially trace elements and vitamins, in both acute and chronic diseases [102]. These micronutrients play crucial roles in metabolic, antioxidant, endocrine, inflammatory, and immune responses during severe illness [104]. It is strongly recommended to include trace elements, such as zinc (Zn), selenium (Se), copper (Cu), and vitamins (A, D, C, and E), from the first day of parenteral nutrition [2,3]. Additionally, providing less than the enteral formula volume required to meet dietary reference intake levels [103] may lead to insufficient intake of vitamins, minerals, and trace elements [42].

However, the direct relationship between micronutrient levels and clinical outcomes remains unclear, limiting the ability to recommend supplementation through enteral nutrition. Clinical symptoms often overlap with those of critical illness, and micronutrient levels can drop significantly during inflammation [105]. Considering the recent trend toward lower-dose artificial nutrition and the avoidance of early parenteral nutrition, it is important to ensure adequate micronutrient intake to prevent deficiencies. Future research should focus on identifying individual baseline requirements by refining the assessment of actual losses and micronutrient status. Due to the limited evidence available, the ESPGHAN/ESPEN/ESPR/CSPEN/ASPEN guidelines, based on expert consensus, recommend using individualized micronutrient products for critically ill children [106]. Commercial products are preferred to reduce the risk of microbial contamination and compounding errors [107].

## 9. Nutritional Support

Providing nutritional support for PICU patients is essential for ensuring adequate energy intake and promoting positive outcomes. Considering that children are in a critical phase of growth and development, their nutritional needs are higher than those of adults, and they vary depending on their growth stage [108]. Additionally, children have lower percentages of lean and fat body mass, making them more susceptible to protein depletion. This increased vulnerability puts them at a higher risk of malnutrition, especially during critical illness [17]. Such conditions often involve severe, acute inflammation that accelerates protein breakdown and causes metabolic imbalances, leading to malnutrition [17]. As the patient’s metabolic and endocrine responses change throughout hospitalization, nutritional support must be adjusted accordingly, with different phases of illness requiring tailored approaches. In response to these challenges, ESPNIC recently updated its clinical recommendations on the assessment and nutritional support of critically ill children [3]. Due to limited high-quality research, these recommendations compile current scientific evidence and, in areas lacking data, provide best-practice guidelines.

### 9.1. Underfeeding and Overfeeding

Both underfeeding and overfeeding have been linked to poor outcomes in PICU patients [35]. A recent study found that only 12.4% of mechanically ventilated children received appropriate nutrition, while 34.3% were underfed and 53.3% were overfed. Another prospective observational study reported that 54% of patients experienced underfeeding due to interruptions in enteral nutrition, compared to 15% without such interruptions [109]. Malnutrition and macronutrient deficiencies during critical illness are associated with increased morbidity, including infections, weakness, prolonged mechanical ventilation, delayed recovery, and higher mortality rates [81]. On the other hand, overfeeding during the acute phase can also be harmful, leading to increased VO_2_ and VCO_2_, difficulties in weaning from the ventilator, lipogenesis, liver dysfunction, and metabolic syndrome [110]. Recent RCTs have demonstrated that early full nutritional support does not benefit critically ill patients and may even cause dose-dependent harm [10,40].

### 9.2. Refeeding Syndrome

When medical nutrition is reintroduced after a prolonged period of starvation, there is an increased metabolic demand and intracellular transport of micronutrients and electrolytes [111]. This can reveal preexisting deficiencies and trigger life-threatening symptoms. Rapid refeeding in severely malnourished children can result in sudden fluid shifts and disruptions in electrolyte balance, leading to conditions such as hypokalemia, hypophosphatemia, hypomagnesemia, hyponatremia, and hypoalbuminemia [111]. These imbalances are often associated with cardiac, neurological, neuromuscular, pulmonary, and hematological complications, with a high risk of mortality. However, diagnosing refeeding syndrome in critically ill children is challenging due to frequent confounding factors affecting electrolyte levels [112]. The hallmark of this syndrome is refeeding hypophosphatemia, defined as a drop in phosphate levels below 0.65 mmol/L within 72 h of starting nutrition, caused by the intracellular uptake and incorporation of phosphate into energy-rich bonds [113].

When refeeding hypophosphatemia occurs early in critical illness, temporarily limiting nutrition intake while correcting vitamin and electrolyte deficiencies may be beneficial, as demonstrated in the Refeeding RCT [114]. To prevent refeeding syndrome, it is advisable to ensure adequate micronutrient intake in all patients, which may require parenteral administration of micronutrients and electrolytes, particularly in the acute phase of illness [98]. Additionally, prolonged underfeeding should be avoided, and nutritional targets should be individualized during the initial days after ICU admission [11].

## 10. Enteral Nutrition

Enteral nutrition (EN) is the preferred method for feeding critically ill children [17]. Research has shown that EN is both feasible and safe for pediatric patients with various medical and surgical conditions, including those on vasoactive medications [2,115,116]. EN offers significant physiological advantages by utilizing the natural gastrointestinal route, which stimulates protein synthesis and supports the interaction of nutrient substrates with hormones, such as gastrin, enteroglucagon, growth hormone, and epidermal growth factor, thereby maintaining nutrient absorption regulation. Additionally, EN helps preserve the integrity of the intestinal mucosa, preventing the translocation of toxins and microbes into the bloodstream—a key factor in reducing the risk of multiple organ failure [17]. Compared to parenteral nutrition, EN is also more cost-effective and associated with a lower risk of infection.

**Figure 3:**
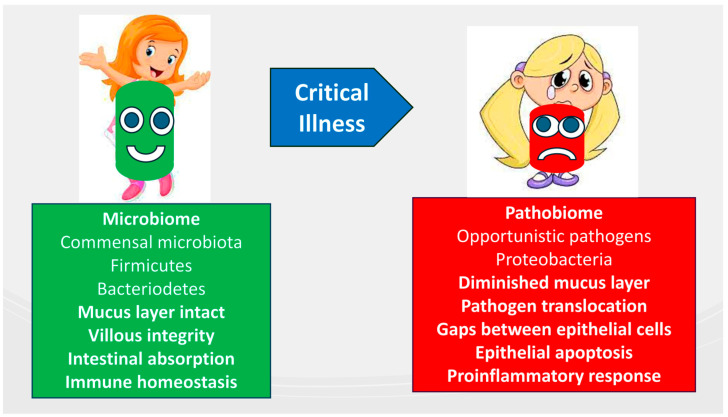
Composition and functions of the intestinal microbiome in critically ill patients compared to healthy children.

EN further supports the preservation of microbiome diversity during critical illness by countering the effects of luminal nutrient scarcity, which can lead to a shift toward a pathogenic microbiome, or pathobiome. This preservation is essential for maintaining villous integrity, facilitating intestinal absorption, and supporting immune homeostasis [117]. Additionally, the gut microbiota affects the interactions between the central and enteric nervous systems, known as the gut–brain axis [118], by connecting the brain’s emotional and cognitive centers with the functions of the peripheral intestines [119]. The gut–lung axis has recently been recognized as a key factor in the pathophysiology of ARDS, emphasizing the bidirectional communication that involves bacterial and immune interactions between the gut and lungs within their respective environments [120]. A pathobiome, characterized by reduced Firmicutes and Bacteroidetes and increased Proteobacteria, is associated with poor revascularization, a compromised mucus layer, and increased pathogen translocation through epithelial gaps. These changes contribute to epithelial apoptosis, impaired nutrient absorption, and pro-inflammatory response, resulting in gastrointestinal disturbances (Figure 3).

### 10.1. Early Initiation and Advancement

ESPNIC recommends starting enteral nutrition within the first 24 h of admission for all critically ill children, unless contraindicated [3]. Large cohort studies have demonstrated that initiating EN within 24 to 48 h of PICU admission and achieving up to two-thirds of the nutritional goal within the first week are associated with improved clinical outcomes [2,3]. In adults, early EN has been linked to significantly lower mortality rates [121,122] and shorter ICU/hospital stays, including among COVID-19 ICU patients [123]. Similarly, in the PICU, early EN initiation within the first 48 h is associated with higher caloric intake, fewer complications, reduced costs, lower mortality, shorter hospital stays, and less weight loss compared to delayed EN [124]. A recent multicenter study also found that early EN enhances nutrient delivery, shortens mechanical ventilation duration, and reduces the incidence of constipation in critically ill children [125].

### 10.2. Specific Situations

International guidelines support the early initiation of enteral nutrition (EN) in critically ill children who have stabilized after receiving hemodynamic support and other interventions [2,3,66]. The recommendation is to gradually increase EN until the child’s nutritional needs are met, using step-by-step algorithms and bedside monitoring to detect and manage EN intolerance while optimizing delivery rates [2,115]. According to recent guidelines, early EN is also recommended for stable children on extracorporeal life support (ECLS), term neonates and children stable on pharmaceutical hemodynamic support, and those recovering from cardiac surgery [3]. Critically ill neonates with umbilical arterial catheters or those on PGE1 infusion should receive EN with appropriate monitoring [2,3]. Early EN is safe and feasible in pediatric patients with mild-to-moderate acute pancreatitis and has been associated with improved outcomes [126]. Additionally, a comprehensive nutritional assessment and individualized approach may lead to better clinical and nutritional outcomes for patients with COVID-19 [127], who often remain in a prolonged acute, catabolic phase [128].

### 10.3. Contraindications

In the early stages (~D1–3), energy should be estimated at around 70% of the target and gradually increased to meet the actual REE as the stay progresses [75]. Low-dose protein delivery (<0.8 g/kg/day) can be used early (~D1–2) and progressed to ≥1.2 g/kg/day as patients stabilize, with consideration of avoiding higher protein intake in unstable patients and in acute kidney injury not on CRRT. EN should be withheld in patients undergoing active resuscitation or who are hemodynamically unstable. In cases where vasopressor agents are being used, EN should be initiated at low doses [65]. Delaying or slowing the advancement of EN is recommended in situations involving gastrointestinal (GI) bleeding, mesenteric ischemia, GI intolerance, risk of aspiration, intestinal obstruction, abdominal compartment syndrome, refeeding syndrome (or phosphate levels below 0.65 mmol/L), or unresolved hemodynamic instability despite vasopressor support [129]. Conversely, no delay is necessary for patients who are adequately resuscitated (e.g., normalized lactate levels), even if they are on low-dose (<0.1 μg/kg/min) or medium-dose (0.1–0.3 μg/kg/min) norepinephrine equivalents [130], have an open abdomen, are under neuromuscular blockade, receiving therapeutic hypothermia, on ECMO, or in prone positioning [131].

### 10.4. Common Barriers to Enteral Nutrition

Delays in EN are often due to gastrointestinal intolerance or fluid restrictions, leading to insufficient calorie and protein intake [22]. Barriers to EN in PICUs, though primarily identified through surveys, highlight common issues, such as delayed initiation, procedural interruptions, perceived intolerance, prolonged fasting for procedures, and caregiver attitudes [132,133]. EN interruptions occur frequently, particularly in children with severe conditions or prolonged stays, reducing their caloric and protein intake [125].

Traditional reliance on gastric residual volume as a marker of feeding intolerance has been reconsidered, as recent guidelines advise against its routine use [134]. Evidence shows that measuring the gastric residual volume does not reduce the risk of aspiration or pneumonia but leads to unnecessary EN interruptions that can contribute to malnutrition [3]. Additionally, there is insufficient evidence to support the routine use of prokinetics to enhance gastric emptying and EN tolerance [3].

Current guidelines, based on observational studies, recommend minimizing EN interruptions to achieve nutritional goals [2,3]. Proposed strategies include setting minimum EN hold times before interruptions, implementing reduced fasting protocols, and using volume-based feeding strategies to compensate for lost intake [116,124,135]. For patients with fluid restrictions, protein- and energy-dense formulas may be beneficial, particularly for children with heart disease [27]. Moreover, non-invasive ventilation should no longer be considered a barrier to enteral feeding, according to recent evidence [136].

### 10.5. Enteral Feeding Formulas

For most critically ill children, polymeric formulas are recommended as the first-line option for enteral nutrition, unless contraindicated [3]. In cases where fluid restrictions are necessary, protein- and energy-dense formulas may help meet nutritional requirements [3]. These energy-dense formulas can also be useful during the transition to oral feeding, particularly with an intermittent feeding schedule [65]. If polymeric formulas are poorly tolerated or contraindicated, elemental and semi-elemental (peptide-based) formulas may be considered to improve tolerance and support the progression of enteral feeding [3].

### 10.6. Pharmaco-Nutrition

Pharmaco-nutrition or immuno-nutrition formulas containing glutamine, arginine, omega-3 fatty acids, selenium, and zinc are not recommended for critically ill children [3]. Randomized controlled trials (RCTs) in this population [137], including those with septic shock [138] or severe head injuries [139], indicate that while immuno-nutrition may influence cytokine modulation and improve nutritional and antioxidant markers, it does not provide additional benefits over standard early enteral nutrition.

A secondary analysis from the EFFORT RCT showed no correlation between glutamate or glutamine levels and malnutrition indicators or the effectiveness of nutritional support [140]. In critically ill children, glutamine supplementation prolonged the elevation of HSP-70 levels but did not impact IL-10 and IL-6 levels [141]. Moreover, high doses of glutamine did not enhance the production of Th1, Th2, or Th17 cytokines in healthy or septic human PBMCs [142]. Elevated glutamine levels were found to decrease HSP72 levels after LPS exposure without affecting early-induced HSP72 mRNA [143]. Similarly, a randomized, double-blind, multicenter clinical trial examining the enteral administration of L-citrulline versus a placebo in critically ill adult patients on invasive mechanical ventilation, without sepsis or septic shock, found no significant difference in SOFA scores on day seven between the two groups [144].

Omega-3 PUFAs may help decrease the inflammatory response and lower the risk of sepsis, septic shock, and multiple organ dysfunction syndrome in patients with severe burns. They may also contribute to a shorter hospital stay, though they do not reduce the risk of death. However, the evidence is limited due to the quantity and quality of the RCTs reviewed, resulting in a low level of confidence in these findings [145].

While probiotics and fecal microbiota transplantation might aid in restoring the microbiome and improving outcomes, there is limited research available to support these approaches [146].

### 10.7. Route and Patterns of Enteral Feeding

Gastric feeding is preferred, as it follows the natural physiological route and is generally as safe as post-pyloric (duodenal) feeding in critically ill children [3]. However, post-pyloric feeding may be appropriate for children at high risk of aspiration or those requiring prolonged fasting for surgeries or procedures. Intermittent feeding, which aligns feeding and fasting periods with regular diurnal patterns, may help reduce circadian rhythm disturbances and activate fasting responses that promote cellular recovery through processes such as autophagy and ketogenesis [147]. Despite these potential benefits, there is no definitive evidence favoring continuous or intermittent enteral feeding over the other. In critically ill patients, an intermittent feeding regimen administered six times per day showed no difference in metabolite patterns over time compared to continuous feeding, whether observed over 24 h or during a ten-day intervention [148]. In critically ill children, there was no significant difference in the time required to reach target calorie and protein levels between the two different enteral nutrition delivery methods [149]. Another study was unable to confirm the feasibility of an overnight fast, as it did not demonstrate non-inferiority in daily caloric intake [150].

## 11. Parenteral Nutrition

In both adult (EPaNIC, n = 4640) [10] and pediatric (PEPaNIC, n = 1440) [151] RCTs, early supplemental parenteral nutrition (PN) was associated with prolonged ICU stays. This approach led to increased dependency on vital organ support and a higher incidence of new infections compared to delaying PN until one week after ICU admission [151]. Large RCTs in adults, including CALORIES (n = 2400) [152], Nutrirea-2 (n = 2410) [153], EDEN (n = 1000) [154], PermiT (n = 894) [155], and TARGET (n = 3957) [40], suggested that the harm observed with early PN in the EPaNIC and PEPaNIC trials was more likely due to the higher nutritional dose rather than the intravenous delivery route. In children, delaying PN for one week in the PICU did not negatively impact survival, growth, health status, or neurocognitive development, and improved inhibitory control two years after PICU admission [156]. Furthermore, avoiding early PN in critically ill children showed no long-term negative effects four years post-randomization, and it even protected against emotional and behavioral issues, supporting the move away from early PN [157]. The discovery of abnormal DNA methylation linked to early PN use in the PICU provides a biological explanation for its long-term negative effects on mood and behavior in critically ill children four years post-ICU admission [158]. Additionally, in adults, early PN increased the incidence of ICU-acquired weakness and delayed recovery from it [78]. Conversely, late PN did not impact two-year survival or physical function in critically ill adults, regardless of their nutritional risk [77].

In light of this recent evidence, the latest European feeding guidelines for critically ill adults have shifted from endorsing early aggressive feeding to advocating for a more conservative approach during the first week of critical illness [129]. Similarly, the 2022 ASPEN guidelines advise against using supplemental PN during the first week of ICU admission in adults [42]. According to the latest ESPNIC guidelines, withholding PN for one week is also recommended for critically ill children and neonates, regardless of their nutritional status, provided that micronutrient needs are met [3]. This shift toward reduced feeding during the acute phase should not increase the risk of refeeding syndrome, which results from deficiencies in micronutrients and electrolytes, such as vitamin B1, potassium, and phosphate [111]. If enteral feeding remains unachievable for an extended period, PN should be carefully administered according to the patient’s calculated needs, with a focus on preventing overfeeding. Essential fatty acids should be provided if PN is needed for more than 10 days [42].

When early PN is necessary, guidelines recommend gradually increasing energy and protein delivery, starting with initial doses of 10–15 kcal/kg or less than 70% of indirect calorimetry-estimated resting energy expenditure (IC REE) and protein at less than 0.8 g/kg/day, with gradual advancement over the first week in the ICU [68]. Maintaining optimal glycemic control and proper catheter care is crucial to minimizing infectious complications associated with PN. Clinical judgment regarding the patient’s metabolic tolerance to dextrose (monitoring glycemic control), lipid emulsions (monitoring serum triglyceride levels), and amino acid dosing is essential for delivering appropriate PN [42].

### 11.1. Autophagy

Autophagy is a vital cellular repair mechanism that helps the body recover from critical illness by eliminating damaged organelles and toxic protein aggregates within cells [159,160,161]. During critical illness, there is often an autophagy deficiency. This process is typically triggered by starvation, oxidative stress, and infection but can be inhibited by nutrient intake, insulin therapy, and possibly other medications [161]. Experimental studies have shown that administering early parenteral nutrition during critical illness can create an autophagy-deficient state in the liver and skeletal muscles [162]. This deficiency leads to increased vacuolation, muscle fiber atrophy, fewer intact mitochondria, reduced respiratory chain activity, and elevated markers of liver damage. Research indicates that early parenteral nutrition, particularly with high doses of amino acids, can suppress autophagy [163], which contributes to liver damage and muscle degeneration [162]. Deprivation of amino acids, a strong trigger for autophagy [164], highlights the negative impact of early or full nutrition during critical illness due to autophagy suppression [78,165]. Consequently, in patients unable to receive sufficient enteral nutrition during the first week of hospitalization, substituting or supplementing with parenteral nutrition has been associated with longer hospital stays and higher mortality rates [10], as seen in studies of critically ill adults and children [166].

### 11.2. Ketones

Secondary analyses of the EPaNIC and PEPaNIC trials revealed that delaying early parenteral nutrition promoted ketogenesis, particularly in critically ill children, which contributed to the positive outcomes of the intervention [167,168]. Emerging evidence suggests that ketones play a protective role in muscle stem cells by enhancing their resilience to cellular stress through signaling pathways, which in turn activates muscle regeneration processes [169]. The extent of anabolic resistance in critically ill patients varies over time and between individuals, influenced by factors such as inflammation, hormonal changes [12], and the degree of immobilization, regardless of the method of nutrition delivery [93].

## 12. Clinical Guidelines

Clinical guidelines serve to connect research findings with practical applications in healthcare settings. However, they are not always implemented as intended.

### 12.1. Nutrition Protocols

Protocol-driven, personalized nutritional support that optimizes macronutrient intake and prevents micronutrient deficiencies has been shown to improve outcomes in non-critically ill patients [50]. In critically ill children, properly adjusting nutritional needs—both in terms of energy and nutrients—during various phases of illness and under different types of medical support can significantly impact clinical outcomes [4]. Observational studies recommend that an ICU nutrition support team, which includes a specialized dietitian, should be available to ensure timely nutritional assessments and the implementation of nutrition protocols. This approach helps to optimize nutrient delivery and tailor care to individual patients [2]. Comparative research has shown that enteral feeding protocols can enhance the timing of initiation and consistency in administering enteral nutrition to specific patient groups [170].

### 12.2. Implementation Models

A crucial aspect of clinical practice is the use of a nutritional treatment algorithm to determine whether to initiate enteral or parenteral nutrition, which can be applied in daily care [171]. Despite the existence of many clinical guidelines, implementation often faces delays or is incomplete [66]. Factors contributing to these challenges include the clarity of guidelines, professional issues, such as lack of knowledge or time, organizational barriers, and clinicians’ time constraints and skill gaps in applying new practices [66].

To address this, a recognized framework has been proposed to facilitate the implementation of the ESPNIC clinical nutrition guidelines [172]. This framework outlines the roles and responsibilities of different professionals, emphasizing that successful implementation requires an interdisciplinary approach. It provides clear instructions and tools for healthcare providers, including doctors, nurses, dietitians, and pharmacists, to effectively apply these guidelines in pediatric ICUs. The guidelines should be concise, easily accessible, and understandable to bedside staff, with specific roles and responsibilities clearly highlighted to ensure staff can readily identify what is directly relevant to them [66].

### 12.3. Personalized Monitoring

Advancements in computerized information systems now allow for real-time visualization of nutrition delivery, leading to significant improvements in meeting nutritional goals [173]. Additionally, automated nutrition platforms with feeding tubes equipped with multichannel bioimpedance sensors can detect gastro-esophageal reflux during high-flow nasal cannula oxygen therapy and help prevent aspiration in real time [174].

## 13. Focused Research

Future randomized controlled trials should explore the impact of varying doses, pharmacodynamics, routes, and compositions of individual macro- and micro-nutrients on morbidity and mortality across different phases and phenotypes of critical illnesses.

### 13.1. Uncertainties

Despite numerous large-scale RCTs conducted in recent years, uncertainties persist regarding the optimal timing and dosage of nutrition for critically ill patients. Although prolonged underfeeding through avoiding full nutrition likely comes at a price, the time point when anabolic resistance ceases cannot be monitored, precluding true individualized nutrition. As a result, the exact duration of the acute phase remains uncertain. This is evident in the differing recommendations between European and American guidelines, with the former permitting reduced feeding for 3–7 days [175] and the latter for up to 7–10 days [42]. A recent review by Gunst et al. analyzed RCTs on nutrition in critically ill patients, revealing that early full nutritional support, increased amino acid intake, or energy dosing based on indirect calorimetry targeting 100% of energy expenditure did not confer benefits to any specific ICU patient subgroup [13]. The lack of positive outcomes from early feeding is believed to be associated with anabolic resistance, futile amino acid catabolism, and the suppression of recovery-enhancing mechanisms, such as autophagy and ketogenesis. Another reason recent nutritional RCTs may not have demonstrated the benefits of early enhanced medical nutrition therapy is the lack of indirect calorimetry. Precise measurement of energy expenditure relies on indirect calorimetry, as predictive equations often fail to accurately estimate energy needs in all patients [175]. However, indirect calorimetry does not measure the amount of endogenous energy production that cannot be suppressed by nutritional intake [13]. Furthermore, the meta-analyses incorporated RCTs with varying designs and co-interventions, making the interpretation of the results more challenging.

### 13.2. Perspectives for Future Research

Future research should prioritize investigating the impact of energy dosages at various stages of illness on key outcomes, such as rapid catch-up growth, long-term metabolic function, and neurocognitive development [171]. Interventions that begin and extend beyond the recovery phase, considering outcomes that occur after discharge from the PICU, should also be explored [13]. Additionally, there is a need to develop individualized approaches that enable real-time monitoring of the anabolic response to nutrition in critical care settings. A recent study investigating the relationship between gastrointestinal biomarkers and the progression of enteral nutrition (EN) revealed that fluctuations in the levels of cholecystokinin, leptin, glucagon, citrulline, and intestinal fatty-acid-binding protein 2 were not linked to the volume of EN administered, which serves as an indicator of feeding intolerance during the initial five days of pediatric critical illness [176]. Novel biomarkers, predictive models, or monitoring devices that can predict and signal the body’s response to nutrition are essential to tailor feeding strategies. This is crucial since feeding resistance and persistent gluconeogenesis are likely dynamic and time-dependent, influenced by the body’s stress response and recovery from severe illness. Such advancements would complement indirect calorimetry measurements during both the acute and recovery phases of critical illness.

### 13.3. Personalizing Nutrition

Personalizing nutrition to align with a patient’s unique metabolic profile, disease type, comorbidities, and body composition represents a crucial future direction, though it will necessitate a significant shift in current patient management practices [177]. Ideally, nutrition should be customized using “ready-to-feed” indicators and markers that show when energy delivery is sufficient, and protein is being effectively incorporated into lean body mass. Further studies are also needed to examine the potential interaction between optimizing amino acid intake during the post-acute phase and the role of exercise [178,179]. Additionally, determining when energy intake is optimal while avoiding overfeeding or underfeeding is essential. The future application of multi-omics technology, combined with bioinformatics and mechanistic studies, holds the potential to uncover new mechanisms and pathways impacted by critical illness, thereby guiding more focused research [180].

In the future, tools such as ultrasound, CT scans, and bioelectrical impedance analysis (BIA) will likely be used to assess nutritional risk and monitor the nutritional response. BIA-based non-invasive body composition assessment can assist dietitians in tailoring dietary plans for critically ill patients [181]. Moreover, advancements in devices designed to measure energy requirements and body composition will help meet nutritional goals [75]. Importantly, there is an urgent need for trials evaluating these technologies to determine the best ways to personalize ICU nutrition and improve outcomes throughout the entire PICU patient journey.

## 14. Conclusions

Severe illness in children leads to significant metabolic and endocrine disruptions that evolve through distinct phases, requiring tailored metabolic and nutritional support. It is essential to assess the nutritional status of all critically ill children upon admission to the PICU and continue monitoring it weekly throughout their stay, as they quickly experience significant muscle mass loss and weakness. Polymeric enteral feeding should begin within the first 24 h of admission, provided there are no contraindications. Current guidelines recommend against the use of supplemental parenteral nutrition during the first week in the ICU. Indirect calorimetry remains the only reliable method for accurately measuring a patient’s energy expenditure in clinical practice, enabling personalized nutritional therapy. Considering the body’s natural energy production, it is advised to meet about 70% of the energy requirements during the first week of PICU admission. During the recovery phase and after discharge from the PICU, supporting catch-up growth and muscle recovery requires a nutritional intake that is at least twice the resting energy expenditure. A gradual increase to a minimum enteral protein intake of 1.5 g/kg/day is necessary to avoid a negative protein balance. Implementing protocol-driven, personalized nutritional strategies that optimize macronutrients and prevent micronutrient deficiencies is crucial. Future research should focus on the effects of energy and protein intake at different stages of illness on outcomes such as rapid catch-up growth, long-term metabolic health, and neurocognitive development. Randomized controlled trials evaluating emerging technologies are needed to identify the most effective methods for personalizing medical nutrition and improving outcomes across the PICU patient journey.

## Supplementary Materials

The following supporting information can be downloaded at: https://www.mdpi.com/article/10.3390/nu16203523/s1. Table S1: Eligibility criteria for Population, Intervention, Comparison, Results, and Study Design (PICOS). Table S2: Summary of assessed bias risk for the randomized studies included in current guidelines [2,3,42,66,68,81,99,103,121,129,175] that support this study. Table S3: The GRADE method used by current guidelines included in the present study.
