# Supplementary material for: Personalized Nutrition in the Pediatric ICU: Steering the Shift from Acute Stress to Metabolic Recovery and Rehabilitation
Source: Nutrients. 2024 Oct 17;16(20):3523. doi: 10.3390/nu16203523 (PMC11509937; doi:10.3390/nu16203523)
Supplement: Supplementary file 1 [file nutrients-16-03523-s001.zip › nutrients-3197745-supplementary.pdf]

Supplementary material

Table S1. Eligibility criteria for Population, Intervention, Comparison, Results and Study Design (PICOS)

PICOS	Inclusion Criteria	Exclusion Criteria
Population	ICU paediatric and adult	non-ICU Patients
Intervention	Enteral or parenteral nutrition (assessment, dosing, type of nutrition, route, supplements, etc.)	
Comparator	Different types of intervention (early vs. late, high vs. low, acute vs. chronic, stress vs. recovery, etc.)	
Outcomes	Effect of the intervention on mortality, ICU or hospital length of stay, infections, quality of life, or complications	
Study Design	Guidelines; randomized placebo-controlled trials; controlled clinical trials; double-blind, randomized controlled studies; systematic reviews; meta-analyses	Book chapters, notes, comments, conference abstracts, case reports/series, animal or in-vitro studies
Time frame	From 2004 to 2024, with an emphasis on studies published within the last three years	
Geography	Global	
Language	English	

Table S2. Summary of assessed bias risk for the randomized studies included in current guidelines [1–11] that support this study.

Q1	Is it clear in the study what is the 'cause' and what is the 'effect' (i.e. there is no confusion about which variable comes first)?
Q2	Were the participants included in any comparisons similar?
Q3	Were the participants included in any comparisons receiving similar treatment/care, other than the exposure or intervention of interest?
Q4	Was there a control group?
Q5	Were there multiple measurements of the outcome both pre and post the intervention/exposure?
Q6	Was follow-up complete and if not, were differences between groups in terms of their follow up adequately described and analyzed?
Q7	Were the outcomes of participants included in any comparisons measured in the same way?
Q8	Were outcomes measured in a reliable way?
Q9	Was appropriate statistical analysis used?

Table S3. The most recent guidelines included in the present study used the Grading of Recommendations, Assessment, Development, and Evaluation (GRADE) method [1–11].

	Grade Comparisons	Grade Recommendation for Clinical Practice	Grade Evidence	Grade Strength
1	Higher versus lower weight-based energy intake	Clinical outcomes - Safety	Low-High	Weak-Strong
2	Higher versus lower weight-based protein intake	Clinical outcomes - Safety	Low-High	Weak-Strong
3	Indirect calorimetry versus predicted energy expenditure equations	Clinical outcomes - Safety	Low-High	Weak-Strong
4	Feeding protocols versus no protocols	Clinical outcomes - Safety	Low-High	Weak-Strong
5	Early versus late enteral nutrition (EN) in a) adults b) children	Clinical outcomes - Safety	Low-High	Weak-Strong
6	Standard polymeric formula versus specialty formulas	Clinical outcomes - Safety	Low-High	Weak-Strong
7	Pharmaconutrition versus non-immune-modulating EN formulations	Clinical outcomes - Safety	Low-High	Weak-Strong
8	Routine monitor of gastric residual volumes versus clinical signs of EN intolerance	Clinical outcomes - Safety	Low-High	Weak-Strong
9	Gastric versus postpyloric route of EN	Clinical outcomes - Safety	Low-High	Weak-Strong
10	Exclusive isocaloric parenteral nutrition (PN) versus EN	Clinical outcomes - Safety	Low-High	Weak-Strong
11	Early versus late PN in a) adults b) children	Clinical outcomes - Safety	Low-High	Weak-Strong
12	Supplemental PN (SPN) combined with EN versus EN alone	Clinical outcomes - Safety	Low-High	Weak-Strong
13	Mixed-oil lipid injectable emulsions (iles) versus soybean oil	Clinical outcomes - Safety	Low-High	Weak-Strong
14	Fish oil (FO)–containing ILE versus non-FO ILE.	Clinical outcomes - Safety	Low-High	Weak-Strong
15	Supplementary antioxidants and vitamins versus no supplementation	Clinical outcomes - Safety	Low-High	Weak-Strong

References

1. Berger, M.M.; Shenkin, A.; Schweinlin, A.; Amrein, K.; Augsburger, M.; Biesalski, H.-K.; Bischoff, S.C.; Casaer, M.P.; Gundogan, K.; Lepp, H.-L.; et al. ESPEN Micronutrient Guideline. *Clin Nutr* **2022**, *41*, 1357–1424, doi:10.1016/j.clnu.2022.02.015.
2. Compher, C.; Bingham, A.L.; McCall, M.; Patel, J.; Rice, T.W.; Braunschweig, C.; McKeever, L. Guidelines for the Provision of Nutrition Support Therapy in the Adult Critically Ill Patient: The American Society for Parenteral and Enteral Nutrition. *JPEN J Parenter Enteral Nutr* **2022**, *46*, 12–41, doi:10.1002/jpen.2267.
3. Joosten, K.; Embleton, N.; Yan, W.; Senterre, T.; ESPGHAN/ESPEN/ESPR/CSPEN working group on pediatric parenteral nutrition ESPGHAN/ESPEN/ESPR/CSPEN Guidelines on Pediatric Parenteral Nutrition: Energy. *Clin Nutr* **2018**, *37*, 2309–2314, doi:10.1016/j.clnu.2018.06.944.
4. McClave, S.A.; Taylor, B.E.; Martindale, R.G.; Warren, M.M.; Johnson, D.R.; Braunschweig, C.; McCarthy, M.S.; Davanos, E.; Rice, T.W.; Cresci, G.A.; et al. Guidelines for the Provision and Assessment of Nutrition Support Therapy in the Adult Critically Ill Patient: Society of Critical Care Medicine (SCCM) and American Society for Parenteral and Enteral Nutrition (A.S.P.E.N.). *JPEN J Parenter Enteral Nutr* **2016**, *40*, 159–211, doi:10.1177/0148607115621863.
5. Mehta, N.M.; Skillman, H.E.; Irving, S.Y.; Coss-Bu, J.A.; Vermilyea, S.; Farrington, E.A.; McKeever, L.; Hall, A.M.; Goday, P.S.; Braunschweig, C. Guidelines for the Provision and Assessment of Nutrition Support Therapy in the Pediatric Critically Ill Patient: Society of Critical Care Medicine and American Society for Parenteral and Enteral Nutrition. *Pediatr Crit Care Med* **2017**, *18*, 675–715, doi:10.1097/PCC.0000000000001134.
6. Mesotten, D.; Joosten, K.; van Kempen, A.; Verbruggen, S.; ESPGHAN/ESPEN/ESPR/CSPEN working group on pediatric parenteral nutrition ESPGHAN/ESPEN/ESPR/CSPEN Guidelines on Pediatric Parenteral Nutrition: Carbohydrates. *Clin Nutr* **2018**, *37*, 2337–2343, doi:10.1016/j.clnu.2018.06.947.
7. Singer, P.; Blaser, A.R.; Berger, M.M.; Alhazzani, W.; Calder, P.C.; Casaer, M.P.; Hiesmayr, M.; Mayer, K.; Montejo, J.C.; Pichard, C.; et al. ESPEN Guideline on Clinical Nutrition in the Intensive Care Unit. *Clin Nutr* **2019**, *38*, 48–79, doi:10.1016/j.clnu.2018.08.037.
8. Singer, P.; Berger, M.M.; Van den Berghe, G.; Biolo, G.; Calder, P.; Forbes, A.; Griffiths, R.; Kreyman, G.; Leverve, X.; Pichard, C.; et al. ESPEN Guidelines on Parenteral Nutrition: Intensive Care. *Clin Nutr* **2009**, *28*, 387–400, doi:10.1016/j.clnu.2009.04.024.
9. Singer, P.; Blaser, A.R.; Berger, M.M.; Calder, P.C.; Casaer, M.; Hiesmayr, M.; Mayer, K.; Montejo-Gonzalez, J.C.; Pichard, C.; Preiser, J.-C.; et al. ESPEN Practical and Partially Revised Guideline: Clinical Nutrition in the Intensive Care Unit. *Clin Nutr* **2023**, *42*, 1671–1689, doi:10.1016/j.clnu.2023.07.011.
10. Tume, L.N.; Ista, E.; Verbruggen, S.; Jotterand Chaparro, C.; Moullet, C.; Latten, L.; Marino, L.V.; Valla, F.V. Practical Strategies to Implement the ESPNIC Nutrition Clinical Recommendations into PICU Practice. *Clin Nutr ESPEN* **2021**, *42*, 410–414, doi:10.1016/j.clnesp.2021.01.005.
11. Tume, L.N.; Valla, F.V.; Joosten, K.; Jotterand Chaparro, C.; Latten, L.; Marino, L.V.; Macleod, I.; Moullet, C.; Pathan, N.; Rooze, S.; et al. Nutritional Support for Children during Critical Illness: European Society of Pediatric and Neonatal Intensive Care (ESPNIC) Metabolism, Endocrine and Nutrition Section Position Statement and Clinical Recommendations. *Intensive Care Med* **2020**, *46*, 411–425, doi:10.1007/s00134-019-05922-5.
